# Editorial: Digital Tools to Measure and Promote Medication Adherence

**DOI:** 10.3389/fmedt.2021.751976

**Published:** 2021-09-27

**Authors:** Job F. M. van Boven, João A. Fonseca

**Affiliations:** ^1^Department of Clinical Pharmacy and Pharmacology, Medication Adherence Expertise Center of the Northern Netherlands (MAECON), University Medical Center Groningen, University of Groningen, Groningen, Netherlands; ^2^Departamento Medicina da Comunidade, Informação e Decisão em Saúde (MEDCIDS), Faculdade de Medicina da Universidade Do Porto, Porto, Portugal

**Keywords:** medication adherence, drug therapy, drug device combination, eHealth (electronic health monitoring), digital medicine, medication technology, MHealth (mobile Health), medication therapy management (MTM)

Back in 2003, the World Health Organization (WHO) released its influential report “Adherence to long-term therapies: evidence for action.” It highlighted the significant clinical and economic burden of medication non-adherence on both patients and societies. Notably, one of its key take home messages included “Increasing the effectiveness of adherence interventions may have a far greater impact on the health of the population than any improvement in specific medical treatments” (1). Increasing the effectiveness of adherence interventions includes efficient identification of risk groups, effective measurement and monitoring, the provision of personalized interventions and their implementation in daily clinical practice (Figure 1).

**Figure 1:**
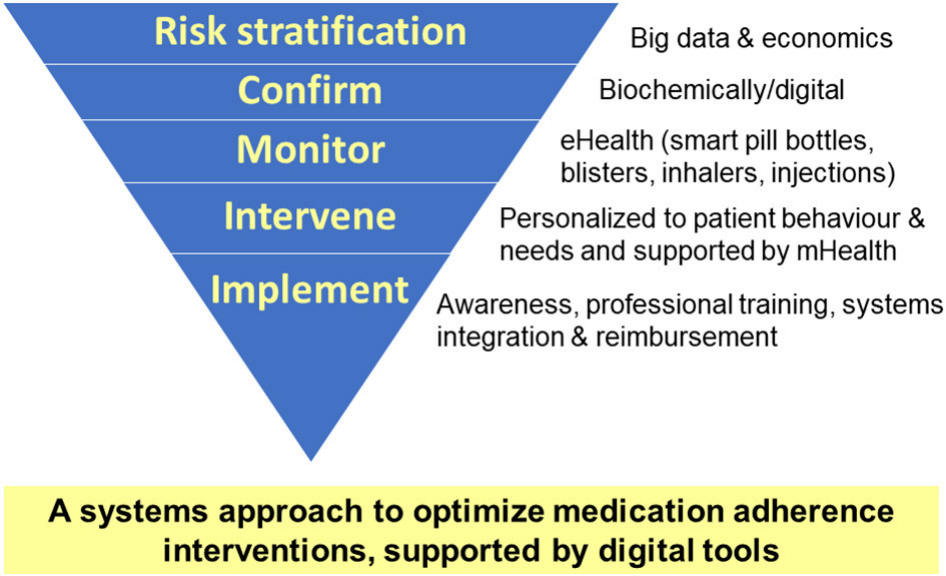
A systems approach to increase the effectiveness of medication adherence interventions.

One of the key steps to increase intervention effectiveness is the use of digital tools to support patients with proper medication use. Indeed, the WHO report included a handful examples of emerging electronic monitoring solutions, yet at that time digital health was still in its infancy. In fact, words such as “ehealth” or “digital medicine” were not even used in this over 200 pages report. It was only since the last decade that we have seen a sharp increase in the number of developed digital solutions to enhance adherence. However, implementation of these solutions in daily clinical care remained low until the world started facing the global COVID-19 pandemic which accelerated the acceptance and uptake of digital care, including remote monitoring solutions of medication adherence. Notably, using remote monitoring we observed an almost 15% increase in adherence to asthma/COPD medication in the months after the outbreak of COVID-19 (2), an increase that is rarely achieved by conventional adherence enhancing interventions in these diseases (3, 4) and beyond (5).

Developments in the field of digital adherence monitoring and management are rapidly evolving and in this special issue of “*Frontiers in Medical Technology*” an overview of different cutting-edge solutions is provided. These solutions range from smartphone applications, to smart inhalers and an electronic auto-injector device, but include also several “how to” challenges related to digital adherence support implementation in daily clinical practice.

In the first study, Backes et al. shine light on the current jungle of apps that claim to support medication adherence and highlighted the marked variation in their quality, which was generally low. They identified over 2,000 apps and only seven fulfilled their inclusion criteria. The authors make clear that proper certification, evaluation, and potential data security issue are key issues to be tackled. Also, long-term clinical studies are required to measure their effects on patient health.

An example of such a clinical study is provided by Jácome et al. In this study, performed across 29 Portuguese secondary care centers a novel app, including an image-based medication detection tool, was evaluated in patients with asthma using inhaled medication. Using this app, patients could take a photo of their inhaler or blister when they took the medicine. The authors conclude that the app was feasible to use however more work is needed to understand higher app usage. Interestingly, this study reminds us that we should not only be aware of medication non-adherence, but also of non-adherence to adherence management solutions.

One way to avoid an additional action such as taking a photo, is combining adherence tracking with natural drug intake behavior by directly linking the drug with the monitoring device, the so called “drug-device combinations” (6). Examples include smart inhalers and electronic auto-injector devices. Brennan et al. and Zabczyk and Blakey both reviewed the evidence of these smart inhalers and found that most devices can enhance adherence, yet several unmet needs remain. Brennan et al. focus on the need for more real-world evidence and more studies on clinical outcomes of these devices, while Zabczyk and Blakey highlight system challenges related to widespread implementation, guidelines, and funding. To enable transdermal drug administration monitoring, a novel electronic auto-injector devices has been developed. Assefi et al. report data of 80 pediatric patients using recombinant human growth hormone therapy and that were previously identified as being non-adherent by such an electronic injection device. Only those with non-adherence received a nurse-led intervention which improved adherence by 9%. This is an interesting example of risk stratification before providing more labor-intensive adherence support.

The final manuscript by Hein et al. relates to the implementation phase of digitally supported adherence management (Figure 1). It entails a structured SWOT analysis of using digital medicine to support personalized adherence management. It combines a literature review with semi-structured interviews with European policy and regulatory stakeholders. Some important issues that are highlighted include the need for further personalization of digital adherence solutions, co-creation of change and optimizing the digital infrastructure of health systems.

Looking forward, persistent challenges related to implementation of digital adherence supporting technology should remain high on policy agendas. These include increasing awareness among stakeholders, as well as easier patient access pathways, integration in clinical support systems and proper reimbursement. On the European level, the European Network of Advance Best practices and technoLogy on medication adherencE (ENABLE) COST Action may address some of these challenges (7), however ongoing efforts from all stakeholders remain necessary. In our daily life, we have already widely embraced digital technology, now it's time for healthcare to follow!

## Author Contributions

JB wrote the first draft. JF commented on it and provided feedback. All authors agreed with submission of the final version.

## Conflict of Interest

The authors declare that the research was conducted in the absence of any commercial or financial relationships that could be construed as a potential conflict of interest.

## Publisher's Note

All claims expressed in this article are solely those of the authors and do not necessarily represent those of their affiliated organizations, or those of the publisher, the editors and the reviewers. Any product that may be evaluated in this article, or claim that may be made by its manufacturer, is not guaranteed or endorsed by the publisher.
